# Osteoarticular Sporotrichosis of the Knee Caused by *Sporothrix brasiliensis*: Two Similar Cases with Different Outcomes

**DOI:** 10.3390/jof9100956

**Published:** 2023-09-23

**Authors:** Bruno Ivanovinsky Costa de Sousa, Livia Cristina Fonseca Ferreira, Marcella Morgado Ramiro de Lima, Juliana dos Santos Barbosa Netto, Guis Saint-Martin Astacio, Andréa Reis Bernardes-Engemann, Priscila Marques de Macedo, Maria Clara Gutierrez-Galhardo, Dayvison Francis Saraiva Freitas

**Affiliations:** 1 Vice-Direção de Ensino, Instituto Nacional de Infectologia Evandro Chagas, Fundação Oswaldo Cruz, Rio de Janeiro 21040-360, RJ, Brazil; 2 Laboratório de Pesquisa Clínica em DST/AIDS, Instituto Nacional de Infectologia Evandro Chagas, Fundação Oswaldo Cruz, Rio de Janeiro 21040-360, RJ, Brazil; 3 Serviço de Imagem, Instituto Nacional de Infectologia Evandro Chagas, Fundação Oswaldo Cruz, Rio de Janeiro 21040-360, RJ, Brazil; 4 Laboratório de Micologia, Instituto Nacional de Infectologia Evandro Chagas, Fundação Oswaldo Cruz, Rio de Janeiro 21040-360, RJ, Brazil; 5 Laboratório de Pesquisa Clínica em Dermatologia Infecciosa, Instituto Nacional de Infectologia Evandro Chagas, Fundação Oswaldo Cruz, Rio de Janeiro 21040-360, RJ, Brazil

**Keywords:** sporotrichosis, *Sporothrix brasiliensis*, osteoarticular, amphotericin B, itraconazole

## Abstract

Sporotrichosis is the most frequent subcutaneous or implantation mycosis in Latin America, and its transmission occurs as a result of traumatic inoculation into the skin by organic matter containing the thermodimorphic fungi of the genus *Sporothrix*. Although cutaneous forms are more common, another important site is the osteoarticular system, whose hematogenous involvement is commonly associated with disseminated forms, especially in people who have an immunosuppressive condition, such as HIV/AIDS, chronic steroid use, and alcohol abuse. We present two cases of osteoarticular sporotrichosis of the knee caused by *Sporothrix brasiliensis* and followed up at our institution, with different outcomes. In the cases presented here, aging, anatomical sites, comorbidities, subtherapeutic serum levels, low adherence to treatment, and late diagnosis for different reasons may explain the observed outcomes. Early diagnosis of *Sporothrix* infection is critical in preventing complications, including death. We also highlight the importance of multidisciplinary follow-up and adherence to treatment for a favorable outcome.

## 1. Introduction

Sporotrichosis is the most frequent subcutaneous or implantation mycosis in Latin America caused by the thermodimorphic fungi of the genus *Sporothrix*. It occurs worldwide, but it is endemic mainly in tropical and subtropical regions [1,2].

While its classical transmission is via traumatic inoculation into the skin or mucosa by organic matter with *Sporothrix* [3,4], in Brazil, especially in the metropolitan region of Rio de Janeiro, *Sporothrix brasiliensis* is the main agent of zoonotic transmission, occurring as a result of scratching, biting, or sneezing from cats with the disease [1,5,6,7,8,9]. This form of transmission, initially considered an outbreak, began in Rio de Janeiro in the 1990s, primarily related to domestic and feral cats, and mostly affecting people from deprived conditions, such as women, retirees, and children who care for and play with these animals [1]. The sustained high number of cases led to a hyperendemic stage of the disease, which further spread to other states and even neighboring countries [1,10,11,12].

The hyperendemic zoonotic *S. brasiliensis* infection in Rio de Janeiro has led to the observation that this species is the most virulent of the genus [10,13]. Although cutaneous forms are the most common clinical presentations, another important site is the osteoarticular system, with involvement of one or more joints or bones [14].

Although osteoarticular sporotrichosis is rare (up to 3–4% of cases) [15], this system is the third most affected site after the skin and mucosa. In disseminated disease, there are cases varying from asymptomatic to extremely painful bone or joint involvement, while localized cases commonly present as large-joint painful monoarthritis [1,16].

Concerning bone involvement, the most affected sites include, in this order, the tibia, carpus and metacarpus, ulna, knee and ankle [17]. This can usually be explained by contiguity with skin lesions due to the small barrier between the skin and the osteoarticular system, causing lesions mainly on the extremities and that are usually unifocal [1]. Either via contiguity or via the hematogenous route, osteoarticular involvement in sporotrichosis is generally indolent with nonspecific symptoms like pain, swelling, and functional limitation in joints [1,14]. As well, osteoarticular cases without cutaneous involvement, probably derived from pulmonary foci and hematogenous dissemination, can make early diagnosis harder and delay administration of the appropriate treatment [17,18]. This delay could result in subsequent unnecessary surgery or permanent damage to the joint and its function, increasing patient morbidity and even mortality [3,14,18]. Osteoarticular involvement via the hematogenous route is commonly associated with disseminated forms, mainly in people living with HIV/AIDS (PLHIV), but it can also be observed in other immunosuppressive conditions, such as chronic steroid use and alcohol abuse [1,4,14,16,17].

The Instituto Nacional de Infectologia Evandro Chagas, Fundação Oswaldo Cruz (INI/FIOCRUZ), located in Rio de Janeiro, is a Brazilian reference institute for the treatment and research of infectious diseases, including sporotrichosis and other endemic mycoses. We present two cases of osteoarticular sporotrichosis of the knee caused by *S. brasiliensis* followed at this institution with different outcomes.

## 2. Patients and Methods

### 2.1. Patient 1

**Figure 1:**
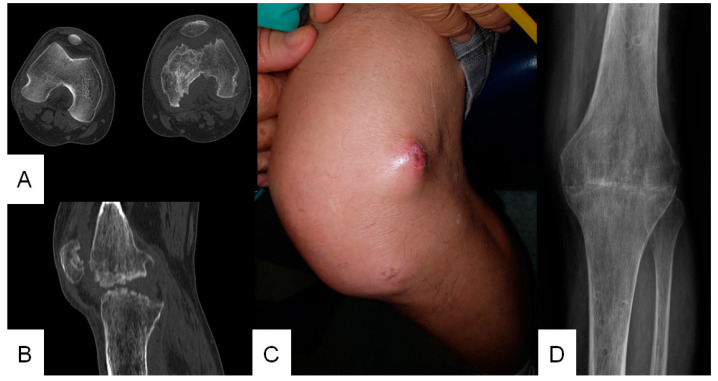
Knee osteoarticular sporotrichosis. (**A**,**B**) Computed tomography scan showing destructive osteoarthritis on the left knee (September 2018). (**C**) Left knee with a nodular sporotrichosis lesion (July 2018). (**D**) Radiograph of the left knee, post-arthrodesis, revealing degenerative joint disease and femorotibial ankylosis (August 2020).

A 66-year-old man presented to INI/FIOCRUZ in June 2015 reporting scratching on his first left finger by a domestic cat diagnosed with sporotrichosis two months before. He progressed with erythema in the left forearm and arthralgia in the right knee. He reported use of amoxicillin and clavulanic acid, initiated after prior medical care at another health unit due to a presumed diagnosis of bacterial infection, but experienced no improvement. There was a medical history of arterial hypertension and type 2 diabetes mellitus, both controlled with medication. Oral itraconazole at a dose of 100 mg/day was initiated. After three months of treatment, he had no cutaneous lesions or arthralgia and was discharged from medical follow-up in September 2015. In July 2017, he returned to INI/FIOCRUZ with a history of left knee pain starting 9–12 months after discharge, which was under investigation with an orthopedist. His family reported daily alcohol consumption and frequent doses of intramuscular and oral dexamethasone to control the knee pain. He had recently undergone a computed tomography scan of the left knee, showing destructive osteoarthritis (Figure 1A,B), and a synovial biopsy with a granulomatous inflammatory infiltrate, from which there was isolation of *Sporothrix* sp. in culture. Oral itraconazole at a dose of 400 mg/day was then prescribed. Ten months later, with no improvement of the symptoms (Figure 1C), a new arthroscopy was performed with collection of synovial fluid and fragments, which yielded *Sporothrix* sp. in culture and a chronic granulomatous inflammation with necrosis, hemorrhage, granulation tissue and fibrosis. Additionally, a post-procedure abscess was drained, and the culture tested negative for bacteria. Intravenous amphotericin B lipid complex at a dose of 200 mg twice a week was added to the treatment with itraconazole, with a cumulative dose of 5.2 g over five months. In March 2019, a culture from the synovial fluid remained positive for *Sporothrix* sp. In August 2019, the patient underwent curettage of the osteoarticular necrotic tissues and arthrodesis when tibia and femur bone fragments also resulted in positive cultures for *Sporothrix* sp. Histopathological analysis from this site revealed a minimal inflammatory process, with no granulomas or fungi. Oral itraconazole at a dose of 400 mg/day was maintained for one more year and was suspended due to cure. A radiograph of the left knee, performed in August 2020 (Figure 1D), revealed degenerative joint disease and femorotibial ankylosis (the latter was secondary to arthrodesis surgery). The patient remains cured of sporotrichosis after three years of follow-up control.

### 2.2. Patient 2

**Figure 2:**
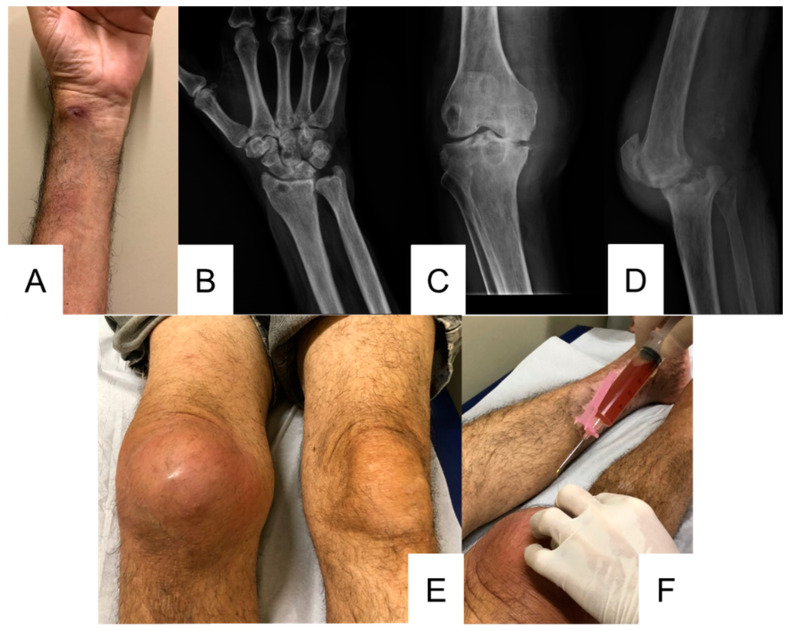
Wrist and knee osteoarticular sporotrichosis. (**A**) Nodule-ulcerative lesion on the left wrist (August 2018). (**B**) Radiograph of left wrist showing osteolytic lesions on the distal radius and capitate (July 2020). (**C**,**D**) Radiographs of the right knee showing tibial plateau erosions, joint space reduction, and irregular osteolytic lesions present on both the femur and tibia (August 2018 and August 2020, respectively). (**E**) Right knee with swelling, erythema, pain, and heat (August 2018). (**F**) Right knee synovial fluid aspiration puncture (August 2018).

A 68-year-old man presented to INI/FIOCRUZ in March 2017 with a 1-year history of a nodular lesion in the right popliteal fossa that spread laterally along with purulent discharge. Three weeks prior to the medical appointment, he also noticed another lesion on the left wrist (Figure 2A). Biopsies of the skin nodule on the knee and the pus from the skin lesion on the left wrist were both positive for *Sporothrix* sp. Radiographs of the left wrist and right knee showed osteolytic lesions, along with reduced joint space and tibial plateau erosions of the right knee (Figure 2B–D). He had type 2 diabetes mellitus with no regular treatment. There was previous contact with healthy cats and gardening activities, but with no trauma reported. In addition, he had an intermittent fever in this period, up to 39 °C, and reported pain in his lower right limb. Itraconazole at a dose of 400 mg/day was initiated in March 2017 for 5 months with a loss of follow-up. In 2018, he returned to the institute with a worsening of the swelling, purulent discharge, and pain in the right knee (Figure 2E), and new clinical specimens were collected (Figure 2F) yielding *Sporothrix* sp. Itraconazole at a dose of 400 mg/day was prescribed again for nearly 5 months and a new loss of follow-up occurred. In July 2020, he resumed follow-up, and his family reported that, since June 2020, there were episodes of “outbreak” (ravings about memories, hallucinations, and seeing people). He had also presented with hyporexia and hypo/hyperglycemia and was not using the medication properly. He was then hospitalized and treatment with an itraconazole and amphotericin B lipid complex was initiated. After a few days, oxacillin was administered due to a methicillin-susceptible *Staphylococcus aureus* growth from the right knee exudate culture. In August 2020, the patient was evaluated by the orthopedics service of another hospital, returning to our institute on the same day. But, after a few hours, he had an episode of cardiopulmonary arrest which was immediately reverted with cardiopulmonary resuscitation maneuvers. Despite the recurrence of spontaneous circulation, the patient’s neurological demotion (Glasgow 3) demanded intubation and transfer to an intensive care unit. Oxacillin was switched to piperacillin/tazobactam for an additional seven days. In the following days, the patient worsened and died.

### 2.3. Mycological Investigation

Clinical specimens were subjected to routine mycological examination at the INI-FIOCRUZ Laboratory of Mycology, which involved culture on sabouraud agar with chloramphenicol (400 mg/L) and mycobiotic agar (Mycosel—DIFCO), incubation at 25 °C, and observation for 4 weeks for fungal growth. Suspected isolates were sub-cultivated on a potato dextrose agar medium (DIFCO) at 25 °C for macroscopic and microscopic morphological studies, and dimorphism was demonstrated by converting to the yeast-like form on BHI (brain heart infusion) agar medium (DIFCO) at 37 °C.

Fungal isolates from both patients were identified as *S. brasiliensis* by a polymerase chain reaction (PCR) for specific *Sporothrix* spp. identification [19], and the in vitro antifungal susceptibility test according to the M38-A2 CLSI guidelines (CLSI, 2008) [20] did not show resistance to itraconazole, posaconazole, ketoconazole (MIC values below 2.0 µg/mL), terbinafine (MIC values below 0.12 µg/mL), or amphotericin B (MIC values below 4.0 µg/mL). These isolates were among the wild-type isolates previously published by Bernardes-Engemann et al. (2022) [6].

## 3. Discussion

The cases presented herein show an unusual form of sporotrichosis, with indolent and aggressive clinical outcomes, and challenges in obtaining a successful medical treatment. Some of the above-mentioned risk factors were present, since both were older patients with diabetes and alcoholism.

In the initial investigation of suspected disseminated cases of sporotrichosis, tracking with simple radiography of all long bones is recommended, in addition to the bones of the hands, wrists, feet and ankles [1,14]. Scintigraphy, computed tomography and magnetic resonance imaging would be more sensitive, but it depends on the availability of each service [1,14]. Images may show cartilage destruction, synovial joint effusion, and osteolytic lesions [1,3,14]. Clinically, pain, edema and functional limitation were observed [1,3,7]. When dissemination is not suspected, but there are signs and symptoms of isolated osteoarticular involvement, targeted imaging should be performed [1,14].

The isolation of *Sporothrix* sp. from joint fluid is the gold standard for confirming diagnosis [1,3,21,22], but the possibility of osteoarticular sporotrichosis and obtaining samples may be problems that can result in late diagnosis. The clinical specimen for culture can be collected from tissue biopsy or synovial fluid. Fungal growth may occur within five days, but sometimes it can take several weeks. Complementary methods can be used, such as polymerase chain reaction and serology [18].

In osteoarticular involvement, the recommended dose of itraconazole is 400 mg/day for at least 6 to 12 months. If the patient is cured, treatment can be suspended, except in PLHIV, which depends on the improvement of the CD4 count (>200 cells/μL). If the patient is not cured, the treatment should be continued until cure is achieved [1,14]. An associated synovectomy may be necessary [1,3]. Amphotericin B is recommended as an initial therapy in cases of extensive disease and in cases refractory to itraconazole [1,3,6]. Preference is given to lipid formulations (3–5 mg/kg/day intravenously) due to their lower nephrotoxic risk [1,7,16]. After clinical improvement, therapy can be switched to itraconazole at a dose of 200 mg twice daily up to resolution [14,16]. Ideally, itraconazole plasmatic level should be monitored, as it has erratic absorption and interacts with several drugs, leading to subtherapeutic levels [1]. In case 1, successful treatment was primarily due to the surgical removal of infected and necrotic tissues, followed by arthrodesis since pharmacological treatment was insufficient. In case 2, after four years of sporotrichosis with multiple follow-up failures, the patient died with osteoarticular sporotrichosis and bacterial sepsis. At INI-FIOCRUZ, we cannot monitor plasma levels of itraconazole, which imposed an important limitation.

Barbaryan and colleagues (2018) reported a case of isolated sporotrichosis of the knee joint in a 33-year-old man without any recognized prior trauma and without skin or other site lesions. The man complained of pain and swelling in his left knee for nine months, and the diagnosis confirmed articular sporotrichosis of the knee without bone involvement. A surgical approach was required for diagnosis and initial treatment with arthroscopy, irrigation, debridement, and major synovectomy. The patient had a history of excessive alcohol consumption as a single comorbidity. He was treated with oral itraconazole at a dose of 400 mg/day with resolution of all symptoms. The authors mentioned the rare presentation and scarcity or lack of yeasts on the histopathology as contributors to late diagnosis and suboptimal treatment leading to morbidity. They also recommended articular sporotrichosis to be included in the differential diagnosis of chronic monoarthritis or polyarthritis in patients with high-risk factors and occupations [23].

Patel and colleagues (2019) reported the case of a 41-year-old male patient with knee sporotrichosis associated with occupational risk and traumatic injury. In this case, there was also a delay in diagnosis. The patient was monitored by the infectious disease department in conjunction with the orthopedic surgery department, and it was decided to proceed with arthroscopy and surgical debridement of the joint after a few months without resolution with oral itraconazole (400 mg/day). There was also a need to change antifungals, to perform sessions of physical therapy, and to use oral non-steroidal anti-inflammatory drugs. Follow-up radiographs showed resolution of the lesion, with images compatible with sequelae. The authors emphasized the importance of early diagnosis and treatment [3].

Sendrasoa and colleagues (2021) showed a case with better outcomes and easier management without the need for a surgical approach. The patient was a 34-year-old man, without comorbidities, who had suffered trauma with plants six months before diagnosis. *Sporothrix schenckii sensu stricto* was isolated and identified via PCR, and the patient was cured after 12 months of oral itraconazole (400 mg/day), with subsequent follow-ups over four years. Infection of the knee joint occurred by local spread from the skin of the leg. The authors highlighted the time until diagnosis, which was shorter than that reported in previously published cases. Possibly, due to this relatively early diagnosis and initiation of treatment, there were no extensive destructive changes. They also emphasized the importance of clinical suspicion for early diagnosis and treatment [15].

The antifungal sensitivity test showed that the low response to drug therapy was not due to in vitro resistance. Aging, the anatomical sites, comorbidities, subtherapeutic serum levels, low adherence to treatment, and late diagnosis for different reasons may explain the pharmacological failure in both cases. Early diagnosis of *Sporothrix* sp. infection is critical to prevent complications, including death. In these two cases, there was the participation of dermatologists, orthopedists, and infectious disease specialists, as well as other health professionals (psychologists, dietitians, psychiatrists, intensivists, social workers, physiotherapists, mycologists, radiologists, and nurses). We highlight the importance of multidisciplinary follow-up and adherence to treatment for a favorable outcome.

## 4. Key Learning Points

- Sporotrichosis should be considered as a differential diagnosis of infectious osteoarthritis in endemic regions.
- Multidisciplinary follow-up is essential for successful treatment.
- Orthopedic surgery should be strongly considered as an adjunct to systemic antifungals.
- In vitro antifungal susceptibility is very important to the therapeutic approach for disseminated sporotrichosis. However, it does not guarantee a clinical response as it involves clinical, immunological, and anatomical particularities.

## Data Availability

All available data are present in the paper. Additional data are unavailable due to privacy or ethical restrictions.
